# Expression of aberrant markers in monitoring of measurable residual disease in B-cell precursor acute lymphoblastic leukemia patients during remission-inducing therapy phase

**DOI:** 10.3389/fonc.2025.1641088

**Published:** 2025-08-27

**Authors:** Ł. Słota, Ł. Sędek, M. Lejman, B. Perkowski, A. Lasia, J. Bulsa, W. Młynarski, J. Kowalczyk, T. Szczepański

**Affiliations:** ^1^ Department of Pediatric Hematology and Oncology, Medical University of Silesia, Katowice, Poland; ^2^ Department of Microbiology and Immunology, Medical University of Silesia, Katowice, Poland; ^3^ Department of Pediatric Hematology, Oncology and Transplantology, Medical University of Lublin, Lublin, Poland; ^4^ Department of Pediatrics, Oncology and Hematology, Medical University of Łódź, Łódź, Poland

**Keywords:** flow cytometry, measurable residual disease, B-cell precursor acute lymphoblastic leukemia, childhood acute leukemia, antigen expression

## Abstract

**Introduction:**

B-cell acute lymphoblastic leukemia (BCP-ALL) is characterized by an abnormal proliferation of immature cells in bone marrow. Leukemic blasts at diagnosis exhibit a so-called leukemia associated immunophenotype (LAIP), which is further used for determination of measurable residual disease (MRD) levels at particular time points during the therapy. Nevertheless, in some patients LAIP proves insufficient for discrimination of blasts from their normal counterparts, therefore, search for novel, aberrant markers is essential. A crucial requirement for these antigens is their expression variability throughout the entire treatment monitoring period.

**Methods:**

The aim of the study was to assess the expression level of four markers: CD66c, CD304, CD72 and CD86 on leukemic cells at diagnosis and at day 15 and 33 of treatment to compare stability of their expression. We also correlated the results obtained with the most common genetic aberrations identified at the diagnosis of BCP-ALL, such as: hyper- hypodiploidy, *BCR::ABL1*, *KMT2A::AFF1*, *ETV6::RUNX1*, *TCF3-rearangement*, *TCF3::PBX1*, *TCF3::HLF*, *KMT2A* and *IKZF1* mutations.

**Results:**

In more than 90% of patients, CD86 overexpression on blast cells was proven at day 15 of treatment and in almost 93% during the entire remission-inducting therapy (day 33). Similarly high positivity rate on leukemic blasts was found for CD72 antigen, which at day 15 was positive in 82% of patients and dropped to 43% at day 33 of treatment. We also found a correlation between the presence of hyperdiploidy and *ETV6::RUNX1* and changes in the expression of particular markers.

**Discussion:**

The obtained results demonstrate that CD86 and CD72 can be successfully used as additional markers for MRD assessment in BCP-ALL.

## 1 Introduction

B-cell acute lymphoblastic leukemia (BCP-ALL) is one of the most common acute malignancies in pediatric patients. It is characterized by abnormal proliferation of immature cells in bone marrow (BM) which results in the accumulation of leukemic cells (blasts), and shortage of mature, immunologically competent cells in peripheral blood. Leukemic blasts typically show an aberrant immunophenotype, as compared to normal B-cell precursors. Altered antigen expression on leukemic cells allows for effective subsequent monitoring of treatment progress through determination of measurable residual disease (MRD) level using flow cytometry (FC). MRD assessment is a powerful outcome predictor, enabling intra-therapy risk stratification and guiding risk-adapted therapeutic approaches in different hematological malignancies, including BCP-ALL (1–9). A well-composed panel for MRD detection should contain antigens capable of discriminating leukemic cells from their normal counterparts (10, 11).

Abnormal precursor cells are characterized by so-called leukemia associated immunophenotype (LAIP). However, it is estimated that in approximately 5% of patients LAIP determination is insufficient for successful MRD assessment, as the expression patterns of the most commonly evaluated markers on blasts may resemble those observed on their physiological counterparts (12). This is particularly important in advanced treatment time points, when normal, regenerating B-cell precursors can be present in BM. To improve the discrimination efficiency of leukemic blasts, it is necessary to use new markers that are differentially expressed on leukemic and normal precursor cells. The recent years brought enormous advance in this field, however the major problem that remains is the stability of their expression throughout the treatment period which is not always satisfactory (13–18).

Another diagnostic challenge is related to the use of immunotherapy (IT). In the case of BCP-ALL, two CD19-targeted strategies are available; one utilizing bispecific monoclonal antibodies targeting T-cells and CD19-positive B-lineage cells (blinatumomab), and the other chimeric antigen receptor T-cells (CAR-T). These therapies are mainly use as a second line treatment in high-risk patients group, with refractory or relapsed ALL. However, as a side effect of IT, the target molecule CD19 can be partially or completely downregulated. It is estimated that approximately 39% of BCP-ALL patients that relapsed after IT, CD19 on leukemic blasts is negative. This poses a critical diagnostic problem for FC, in which the CD19 antigen is a key marker for determination of leukemic cells, including treatment monitoring by assessment of MRD (19–21).

The aim of the study was to determine the fluctuation of four markers expression: CD66c, CD304, CD86 and CD72 on leukemic blasts in pediatric patients with BCP-ALL, by comparing the expression levels of above mentioned antigens at day 15 and 33 of induction treatment and the diagnosis (day 0). As well as possible correlations between the expression levels of the studied markers and the presence of genetic aberrations identified at diagnosis of BCP-ALL.

## 2 Materials and methods

The study cohort consisted of 223 patients diagnosed with BCP-ALL (median age = 3.1, IQR = 2.0; male/female ratio of 1.28–125 male and 98 female) treated at 16 centers affiliated to the Polish Pediatric Leukemia and Lymphoma Study Group. BM samples were analyzed using FC at the reference center for MRD assessment in Zabrze, at the Medical University of Silesia in Katowice, in accordance with the EuroFlow sample preparation and staining protocols (22, 23). BM samples were collected at the day of diagnosis (day 0) and at the day 15 and 33 of induction treatment and stained with a 10-colour panel of monoclonal antibodies including anti-CD66c (PE; BD Biosciences, San Jose, CA, USA), -CD72 (PE; EXBIO, Praha, Czech Republic), -CD86 (BV-510; BD Biosciences, San Jose, CA, USA) and -CD304 (PE; Biolegend, San Diego, CA, USA) antibodies (**Table 1**).

**Table 1:** 10-colour panel for BCP-ALL diagnosis (A) and follow-up (B).

(A)										
Tube no.	FITC	PE	PerCP-Cy5.5	PE-Cy7	APC	AlexaF700	APC-H7	PacBlue	BV-510	BV-605
1	CD123	CD66c	CD34	CD19	CD22	CD45	CD38	CD20	CD13	CD10
2	TdT	CD304	cyIgM		smIgM		Lambda	Kappa		CD24
3	CD15	CD72	CD34		CD117		CD81	CD9	CD86	
4	CD2	TSLPR			CD33		CD14		CD73	
(B)										
Tube no.	FITC	PE	PerCP-Cy5.5	PE-Cy7	APC	AlexaF700	APC-H7	PacBlue	BV-510	BV-605
1	Syto-16	CD66c	CD34	CD19	CD22	CD45	CD38	CD20	CD86	CD10
		CD304								
		CD72								

The selection of the marker for PE position in follow-up panel was made for each patient individually, depending on its expression level at initial diagnosis.

**Figure 1:**
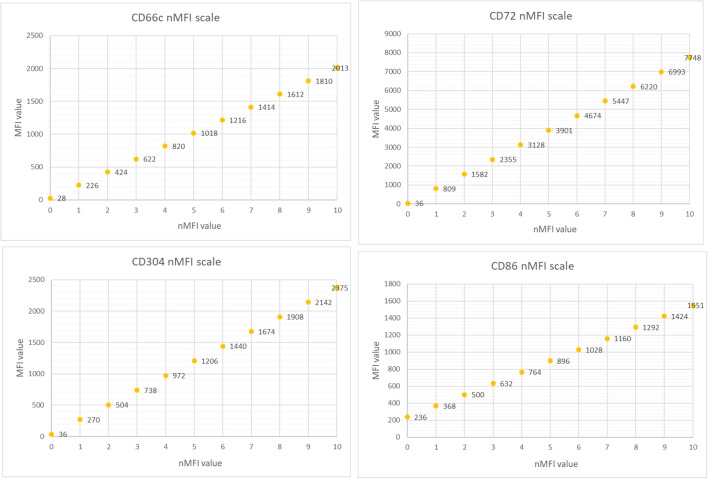
The MFI and the corresponding nMFI values obtained for individual markers.

**Figure 2:**
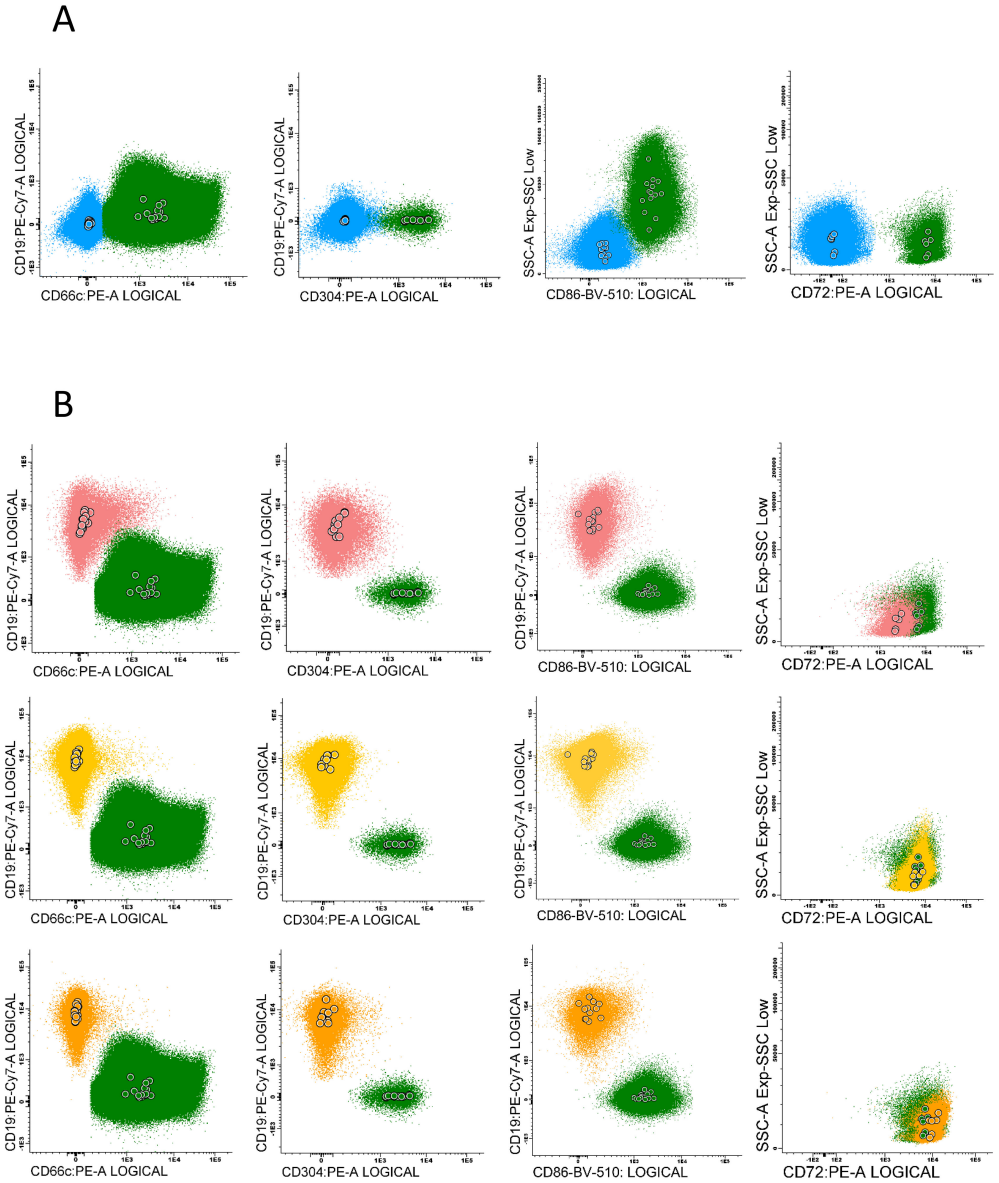
Examples of particular marker expression, presented by positive reference populations for each antigen: CD66c – neutrophils, CD304 – dendritic cells, CD86 – monocytes and CD72 – mature B-cells (green), compared to: **(A)** - the negative populations – mature T-cells (blue). **(B)** - the expression pattern of each antigen on normal B-cells precursors subpopulations (pre-BI - pink, pre-BII - yellow, immature B-cells - orange). Color dots represent the median fluorescence of marker expression for each control sample.

The marker expression levels at different time points were determined by measurement the median fluorescence intensity (MFI). To minimize the subjectivity, we introduced the normalized MFI (nMFI) scale for each evaluated marker (**Figure 1**). The nMFI scale was based on the marker-specific positive and negative reference cell populations, as previously described (24). Briefly, the difference in MFI values between the positive and negative reference populations per marker, was divided into 10 equal intervals. nMFI scores of 0–1 corresponded to the lack of expression, nMFI of 2–6 was considered as low positivity, and nMFI of 7–10 represented maximal expression measured on the positive reference population. Possible overexpression of a marker on blast cells might be calculated by extrapolation of the scale beyond the nMFI value of 10. To determine the nMFI scale, non-leukemic bone marrow samples were used (14 samples for CD66c and CD86; 11 samples for CD304; 6 samples for CD72). All control BM samples were collected from pediatric patients for diagnostic purpose, to exclude proliferative hematological malignancies. As positive reference populations for CD66c, CD304, CD72 and CD86, neutrophils, dendritic cells, mature B-cells and monocytes were used, respectively. As negative reference, the T-cell population was used for each marker. We also assessed the nMFI values of the studied markers on normal B-cell precursor populations (pre-BI, pre-BII and immature B-cells). The results were used to determine the cut-off value discriminating the blast cells from their normal counterparts for each tested marker (**Figure 2**).

**Figure 3:**
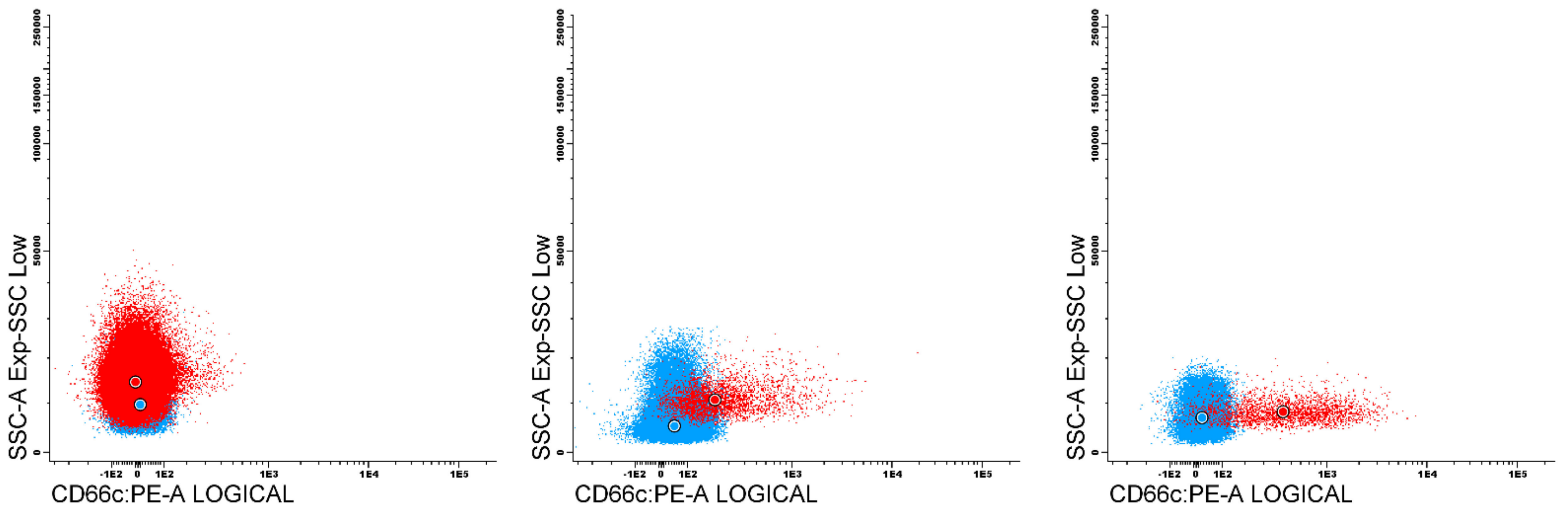
Dot plots presents an examples of leukemic populations (red) against reference negative (blue) for CD66c expression. First plot contain blast population with negative expression of CD66c (nMFI 0), second with nMFI level equal to 1 unit, compared to 2 nMFI on third plot.

The expression of the tested antigens in nMFI units was determined on blast cells at the day of diagnosis (day 0), and at day 15 and 33 of the therapy in MRD-positive patients. The expression variability of the studied markers was evaluated in patients with positive expression of particular marker at day 0, by comparing the nMFI values at different time points for each patient. MRD analysis is strongly based on visual assessment of antigen expression on leukemic blasts which includes natural spread of fluorescence intensity beyond particular median value, especially in cases of heterogeneous marker expression. This means that slight differences in nMFI values (0 – 1) can result from this natural spread of fluorescence. Therefore, we assumed positive expression beginning from 2 nMFI units, which corresponds to the visual assessment with classical dot plots, and allows for complete discrimination of positive population from the remaining cells (**Figure 3**).

The marker expression determined in nMFI units was further correlated with genetic aberrations identified at diagnosis of BCP-ALL, such as: hyperdiploidy and hypodiploidy (standard G-banding technique); *BCR::ABL1* (FISH: LSI BCR/ABL Dual Color, Dual Fusion Translocation Probe, Vysis), *KMT2A::AFF1* (FISH: LSI MLL BA, Metasystem; *MLLT4/KMT2A* Translocation, Dual Fusion, Cytocell), *ETV6::RUNX1* (ETV6/RUNX1Dual Fusion/Translocation FISH probe Kit; Cytotest), *TCF3 rearrangement, TCF3::PBX1, TCF3::HLF* (E2A/PBX1 Plus Translocation, Dual Fusion Probe; CytoCell), other KMT2A rearrangements and IKZF1 mutations (CytoScanHD microarray SNP).

To compare the expression differences between patients with positive and negative aberration, the statistical test was performed. The Shapiro-Wilk test indicated that the majority of expression data series did not fulfill the normal distribution requirements. For this reason, a non-parametric test for independent groups – The Mann-Whitney U test was used.

Patient samples were used following the patient written consent and approval of Bioethics Committee of Medical University of Silesia (PCN/0022/KB1/90/XV/20/21 from 02.02.2021).

## 3 Results

### 3.1 CD66c and CD304

The expression of CD66c and CD304 antigens on leukemic cells was determined at diagnosis in 58 and 52 patients, respectively. CD66c was positive (nMFI ≥2) in 29/58 (50%) of patients while CD304 in 34/52 (65%) of patients. The overall expression level of both markers at diagnosis was relatively low and reached 1.7 ± 9.4 and 3.8 ± 6.3 nMFI units for CD66c and CD304, respectively. However, among MRD-positive cases, the median nMFI at day 0 for CD66c reached 9.4 ± 11.1 and 4.8 ± 6.8 for CD304. Overexpression (nMFI>10) rate for these markers was observed in 11/58 (19%) and 7/52 (13%) patients for CD66c and CD304, respectively (**Table 2**).

**Table 2:** Number and percentage of patients showing different expression levels (in nMFI units) for each tested marker.

time point	n total	Negative		Positive					
		0–1 nMFI		2–6 nMFI		7–10 nMFI		>10 nMFI	
CD66c									
Day 0	58	29	50%	14	24%	4	7%	11	19%
Day 15	58	46	79%	8	15%	2	3%	2	3%
Day 33	15	9	60%	6	40%	0		0	
CD304									
Day 0	52	18	35%	20	39%	7	13%	7	13%
Day 15	52	32	61%	14	27%	3	6%	3	6%
Day 33	15	12	80%	3	20%	0		0	
CD72									
Day 0	82	22	27%	51	62%	9	11%	0	
Day 15	82	17	21%	50	61%	12	15%	3	5%
Day 33	14	10	67%	4	26%	0		1	7%
CD86									
Day 0	85	36	43%	24	28%	13	15%	12	14%
Day 15	85	17	20%	26	30%	20	24%	22	26%
Day 33	23	4	17%	9	39%	2	9%	8	35%

**Figure 4:**
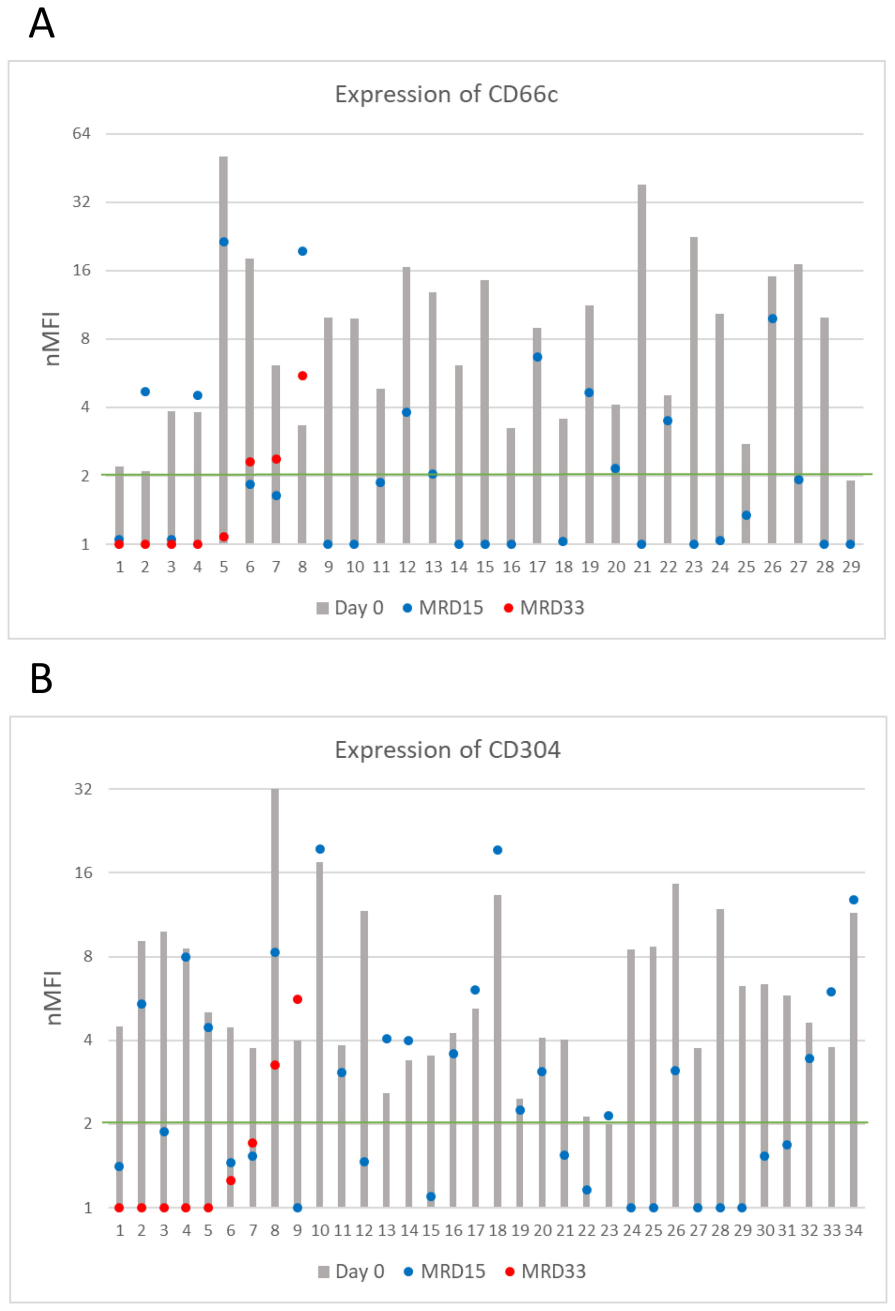
CD66c **(A)** and CD304 **(B)** antigens expression on blasts throughout entire monitoring period (logarithmic scale). The green line represents the cut-off value of positive expression. Only cases positive at day of diagnosis were shown.

Among patients positive at initial diagnosis, at day 15 of treatment the expression level of CD66c remained positive (expression level ≥2 nMFI units) in 11/29 (38%) of patients, but its median expression dropped to 4.7 ± 6.4. Regarding CD304, it remained positive in 18/34 (53%) cases at day 15 of the therapy, but its expression level slightly declined to 4.3 ± 5.2 nMFI (**Table 2** and **Figure 4**). Noteworthy, the expression of CD66c and CD304 turned from negative at initial diagnosis, to low-positive at day 15 of treatment in one patient per each tested marker.

Among patients with positive expression at diagnosis and still MRD-positive at day 33 of treatment (n=8 for CD66c, n=9 for CD304), median expression levels of CD66c and CD304 dropped to 1.1 and 0.9 nMFI units, respectively. The expression remained positive in 1/8 (13%) case for CD66c and in 1/9 (11%) for CD304 during the entire course of induction therapy. Noteworthy, the expression of CD66c and CD304 re-increased at day 33 after having dropped below the positivity cut-off value on day 15 in two and one patient, respectively (**Figure 4**).

### 3.2 CD72

CD72 expression at the day of diagnosis was positive in 60/82 (73%) cases, however its overall median level at day 0 was low, reaching 2.7 ± 2.1 nMFI and 3.4 ± 1.9 nMFI units for the entire patient group, and for CD72-positive cases, respectively. Among CD72-positive patients, a significant increase of CD72 expression level at day 15 of therapy was observed, as compared to the diagnosis (4.2 ± 4.1 vs. 3.4 ± 1.9 nMFI units) which remained positive in 49/60 (82%) cases. Remarkably, in 15 patients, expression increased form negative to positive at day 15 of treatment, making the number of positive patients at follow up higher than at day 0 (n = 67 vs. 60, respectively).

**Figure 5:**
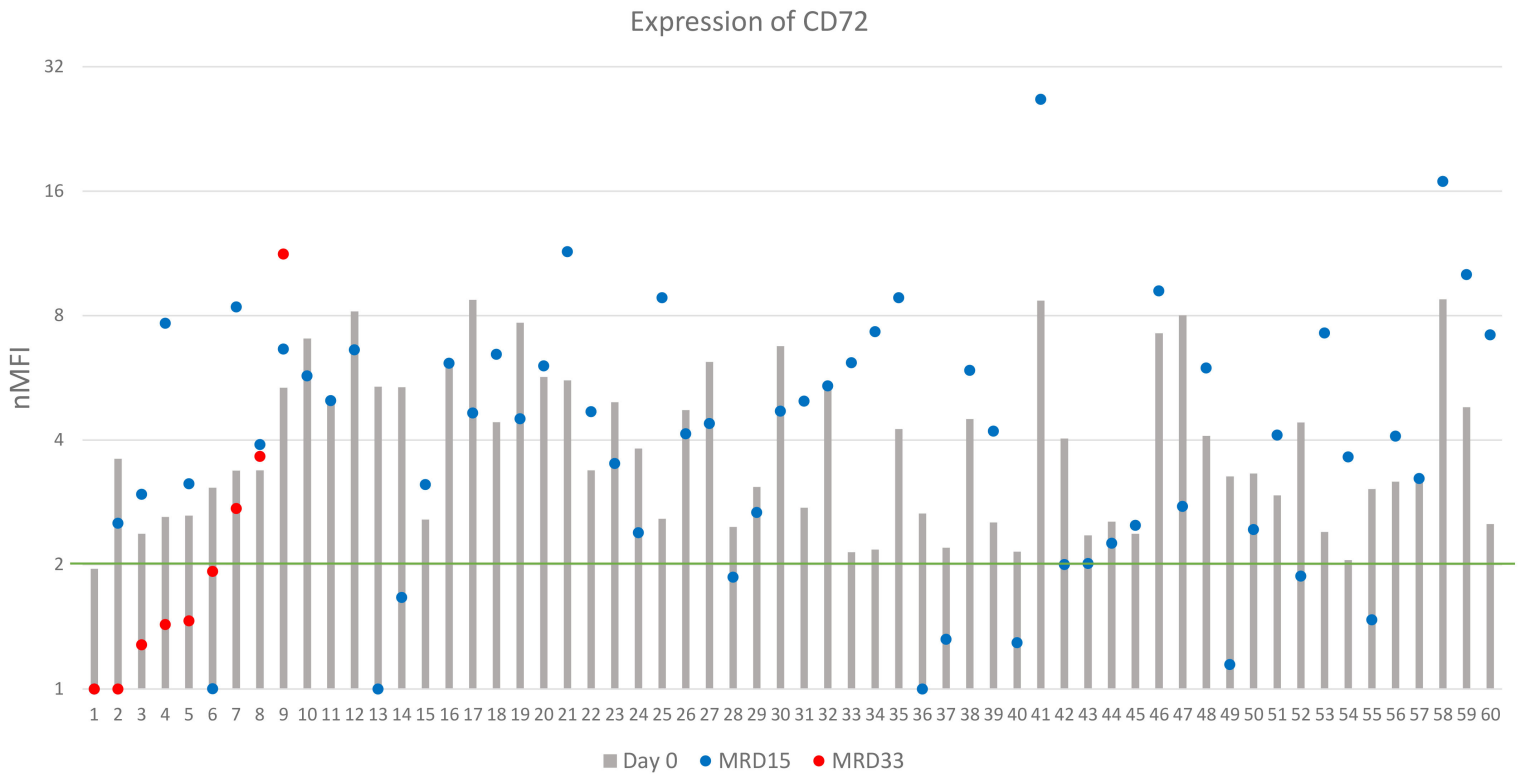
CD72 expression on leukemic cells (logarithmic scale). The green line represents the cut-off value of positive expression. Only cases positive at day of diagnosis were shown.

In 15 cases MRD-positive at day 33, median CD72 expression was equal to 1.5 nMFI units, as compared to 2.4 at day 0. In 5/15 cases, an increase in expression of median value of 1.6 nMFI was observed. In 10/15 patients the expression was lower as compared to the diagnosis, however median value of decrease was only 1.2. Among patients with positive expression on the day of diagnosis (n=7) in three (43%) of them the expression remained positive throughout the entire treatment period (**Figure 5**).

### 3.3 CD86

Expression of CD86 was determined in 85 patients of which 49 (58%) showed positive expression at diagnosis. The overall median nMFI value reached 3.1 ± 4.6, compared to 6.1 ± 4.0 for the CD86-positive patients. Overexpression of CD86 was observed in 12 (14%) patients (**Table 2**). At day 15 of therapy, expression of CD86 remained positive in 46/49 (94%) patients. The median nMFI value for the entire group increased to 6.0 ± 10.4, while in the CD86-positive patients to 8.0 ± 8.1, compared to the day of diagnosis.

**Figure 6:**
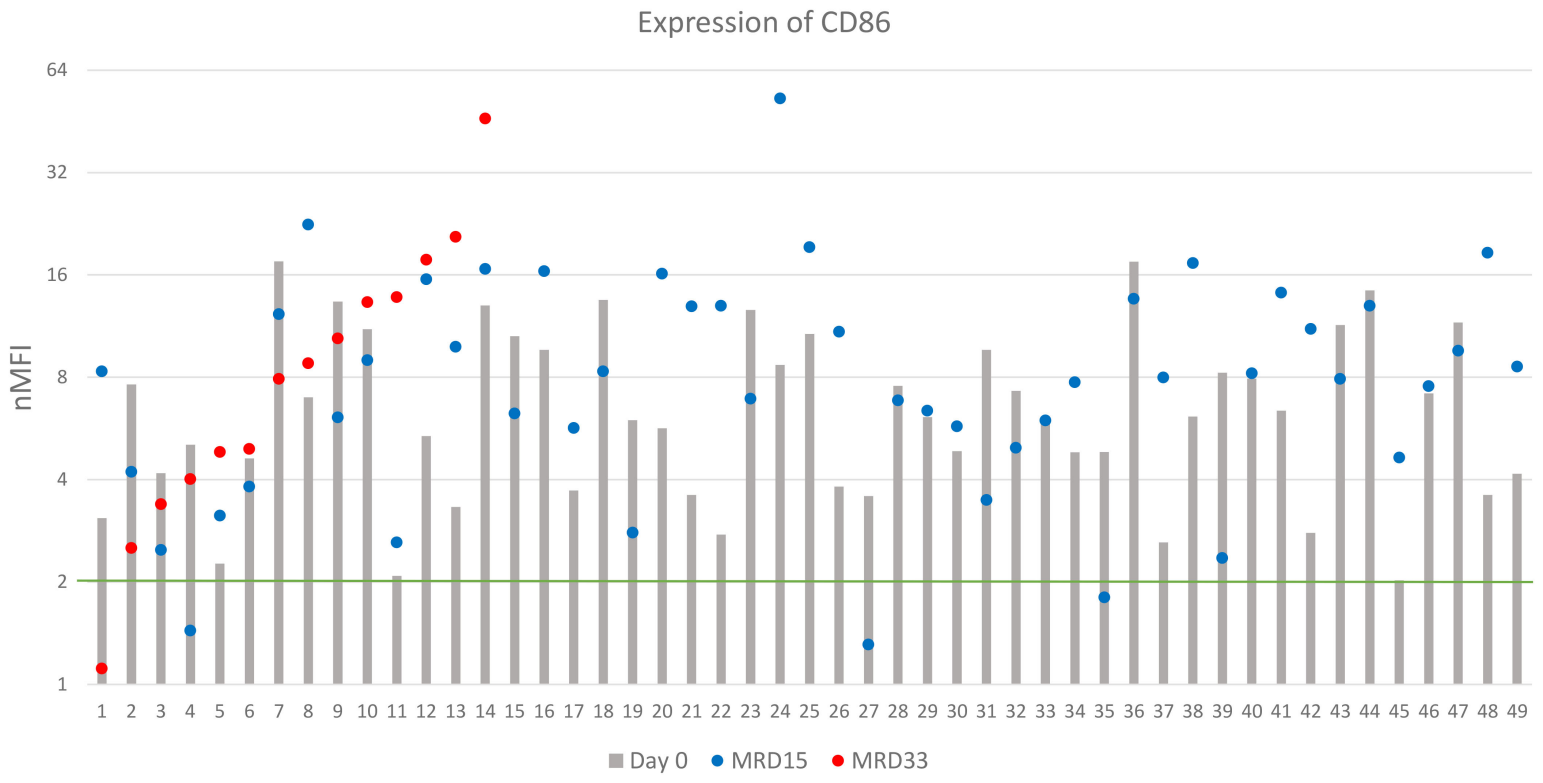
CD86 expression (logarithmic scale). The green line represents the cut-off value of positive expression. Only cases positive at day of diagnosis were shown.

Among 23 patients MRD-positive at day 33 of treatment, median value of CD86 expression was equal to 4.8 nMFI. In 15/23 cases, a significant increase in CD86 expression was observed, with an average value of 9.7 nMFI units, while the decrease (with median decrease rate of 3.3 units) was observed in 7/23 patients, compared to the expression level at day 0. In 13/14 (93%) patients, who were CD86-positive at the day of diagnosis, expression remained positive throughout the entire monitoring period, reaching median value for this group 8.4 nMFI units (**Figure 6**). Noteworthy, 22/36 patients that were negative for CD86 at the day 0 turned positive at day 15 of treatment (**Table 2**).

### 3.4 Correlation of genetic data with expression changes

We also investigated for the possible correlations between the expression levels of the studied markers and the presence of hyperdiploidy, *KMT2A rearrangements* and *ETV6::RUNX1*. Since the numbers of cases positive for hypodiploidy, *TCF-*rearrangement, *BCR::ABL1*, *TCF3::PBX1*, *TCF3::HLF* and *IKZF1* alterations were less than 5, statistical analysis for them was not performed (**Table 3**).

**Table 3:** Comparison of the genetic data obtained with the median expression values of individual antigens at different time points.

Genetic aberration		CD86			CD66c			CD304			CD72		
		n	Day 0	Day 15	n	Day 0	Day 15	n	Day 0	Day 15	n	Day 0	Day 15
Hypodiploidy	pos	1			2	1.7	9.7	1	4.6	3.4	0		
	neg	78			55	1.9	0.6	35	3.4	1.5	44		
Hyperdiploidy	pos	38	4.9	8.4	19	6.1	0.8	6	1.5	1.6	17	3.6	4.1
	neg	41	0.8	3.1	38	0.3	0.4	36	3.8	1.5	27	2.6	3.1
KMT2A rear.	pos	6	1.4	3.9	2	0.1	0.0	2	0.4	0.2	0		
	neg	73	3.6	6.1	55	2.1	0.6	34	3.5	1.5	44		
ETV6::RUNX1	pos	7	0.0	2.1	7	0.0	0.0	21	3.8	1.5	16	2.3	3.9
	neg	72	3.6	6.3	50	3.0	0.7	15	1.4	2.0	28	3.4	3.8
TCF3::PBX1	pos	1	0.5	3.2	2	0.1	0.1	0			1	1.6	2.4
	neg	78	5.0	6.0	55	2.1	0.6	36			43	3.1	3.9
IKZF1-plus	pos	3	0.3	5.2	4	3.1	2.6	1	11.5	12.8	2	7.9	5.2
	neg	73	3.6	6.0	53	1.1	0.5	35	3.4	1.5	42	2.7	3.6
TCF::HLF	pos	1	0.0	0.1	1	0.0	0.1	0			0		
	neg	78	3.2	6.0	56	2.0	0.6	36			44		
TCF-r	pos	2	2.6	2.6	3	0.0	0.1	0			1	1.6	2.4
	neg	77	3.3	6.1	54	2.2	0.6	36			43	3.1	3.9
BCR::ABL1	pos	1	0.0	2.7	4	3.2	2.2	2	12.4	16.1	1	8.7	4.7
	neg	78	3.2	6.0	53	1.3	0.5	34	3.0	1.3	43	2.7	3.7

A part of obtained data was not statistically tested (grey background).

**Figure 7:**
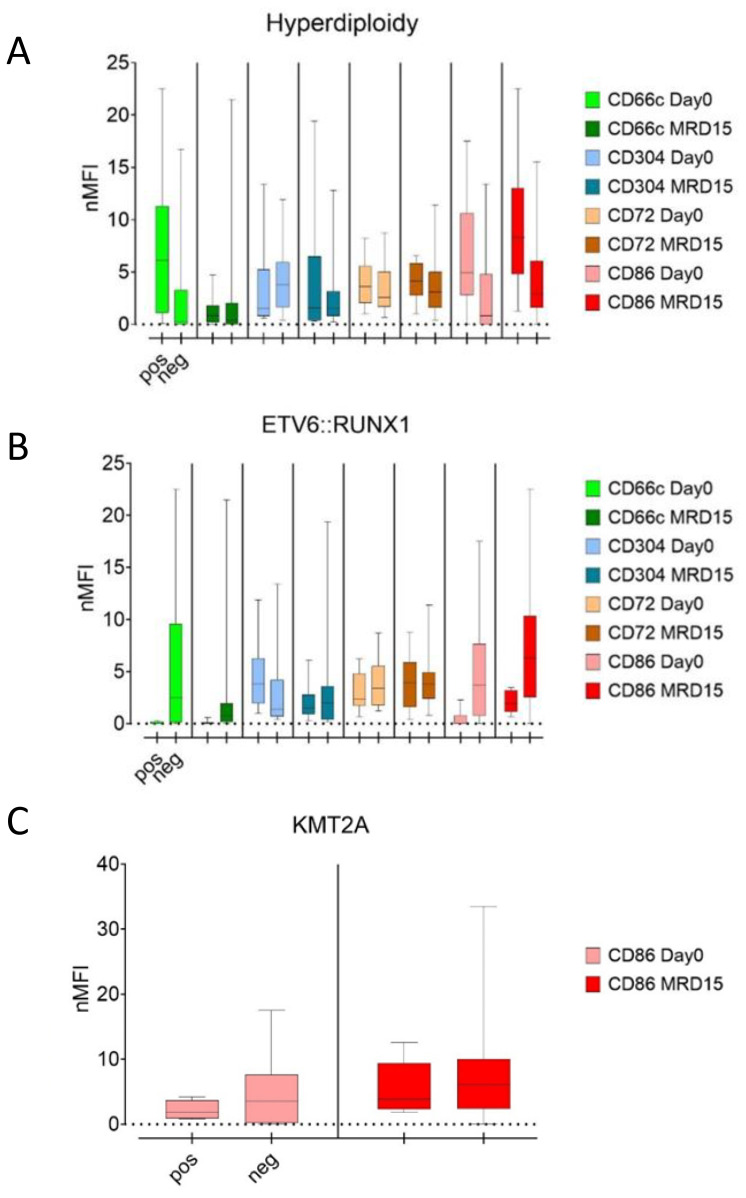
Comparison of median nMFI values in patients with (left bars) and without (right bars) hyperdiploidy **(A)**, ETV6::RUNX1 **(B)**, and KMT2A rearrangements **(C)**.

Hyperdiploidy was the most frequently detected aberrancy, observed in 80 patients. In this group, at the day of diagnosis, significantly higher expression of CD66c and CD86 was observed (p<0.05), in relation to patients without this aberration. Conversely, slightly higher expression of CD304 was found in patients without hyperdiploidy, nevertheless the difference was not significant. Noteworthy, the differences in CD66c and CD304 expression between hyperdiploidy-positive and negative groups vanished during treatment (day 15 and 33), while the level of CD86 was markedly higher throughout the treatment in hyperdiploidy-positive patients. The CD72 expression level was not significantly different between patients with hyperdiploidy and without hyperdiploidy (**Figure 7A**).

In *ETV6::RUNX1*-positive patients, the median expression of CD66c and CD86 antigens did not exceed 1 nMFI unit and was significantly lower than in *ETV6::RUNX1*-negative patients in which CD66c and CD86 reached 3.0 and 3.6 nMFI, respectively (p<0.05). This difference persisted for both antigens over the course of the therapy. In turn, no significant differences in expression of CD86 were observed. For CD304, significantly higher median expression was observed at the day of diagnosis in patients with positive *ETV6::RUNX1* fusion. Nevertheless, this difference was no longer evident at day 15 of therapy. In the case of CD72, no differences in expression were detected either at the day of diagnosis or during treatment (**Table 3**, **Figure 7B**).

In the *KMT2A::AFF1* rearranged group, slightly higher expression level of CD86 was observed for patients without *KMT2A::AFF1* rearrangements, as compared to *KMT2A::AFF1*-rearranged group and this trend persisted through the day 15 of treatment, however statistical significance was not confirmed (**Table 3**, **Figure 7C**).

## 4 Discussion

FC is a powerful diagnostic tool for monitoring of the treatment of ALL patients by MRD detection. A high correlation between MRD level and the outcome of the therapy has been proven. Currently it is one of the most important prognostic factors considered in the treatment of ALL patients (8, 9, 25, 26). The relevance of this technique has also been demonstrated by continuous progress, especially in the search for novel markers and the development of new diagnostic panels to improve the efficiency of leukemic cell detection at improved limits of quantification.

The aim of this study was to determine the expression of selected markers on leukemic cells in patients with BCP-ALL and their expression pattern through the treatment induction phase. The selection of antigens was made based on available literature data reporting the high frequency of overexpression, as well as utility in BCP-ALL monitoring of such markers as CD86 (16, 27), CD66c, CD304 (used in BCP-ALL MRD panel designed by the EuroFlow Consortium) (28, 29) and CD72, a pan-tumor antigen, suggested as a potential key marker for patients with loss of CD19 expression after immunotherapy, and commonly expressed in BCP-ALL (20, 30). In addition, we tried to find any patterns in marker expression reflecting the most commonly detected genetic aberrations in BCP-ALL patients.

The determination of antigen overexpression is very significant in respect to selecting an appropriate diagnostic panel to follow up individual patients. A key feature of a marker useful for MRD detection is the knowledge about its expression pattern and possible fluctuation throughout the treatment monitoring period. In the current study, we demonstrated a persistent overexpression of CD86 and CD72, throughout the whole remission induction treatment, in the vast majority of cases. Of the 49 CD86-positive patients, the expression remained positive in over 90% of cases with the median nMFI value doubling from the day of diagnosis till the day 15 of treatment, and remaining at 1.5-fold higher level until day 33 of treatment, as compared to the day of diagnosis. In our previous study (17), a high stability of CD86 expression at 15 and 33 days of treatment (71%) was reported, which corresponds with the results obtained in the present study. In case of CD304, expression at day 15 of treatment in over half of the patients remained positive which is also in line with the previous findings (17). In turn, Tembhare et al. reported that in post-induction therapy time point, CD86 was positive in 47% of patients, based on visual discrimination of blast cells. In our study, this value was 80% and 83% at day 15 and 33 of induction treatment therapy, respectively. The disparity between Tembhare et al. and our results might be caused by different approaches to interpreting the overexpression, as we decided to rely on numerical data, which are less subjective and independent of the researcher than visual assessment (16).

We also estimated an overexpression level of the examined markers at the day of diagnosis. The highest overexpression rate of 65% and 57% was observed for CD304 and CD86, respectively. This observation is in accordance with the results presented by Tembhare et al., even though different strategies of determination of the cut-off value overexpression were used. Tembhare et al. based on the geometric mean MFI value of exceeding the highest level of normal hematogones, while we took as the cut-off value an expression of equal or more than 2 nMFI (16). Our present results were also similar to those obtained previously, where we reported the frequency of CD86 and CD304 overexpression at diagnosis in 59% and 58% of patients respectively (17). For CD66c, in two-thirds of cases, we found low or no expression (nMFI of 0-6), however in the minority of CD66c-positive patients, its level remained stable until day 15 of therapy in 38% of cases. Similar results were presented by Guillaume et al., who observed CD66c overexpression in 40% of patients, being the most frequently expressed aberrant myeloid marker on blast cells in BCP-ALL (31).

CD72 is considered a potential alternative to CD19 gating marker in BCP-ALL patients in whom anti-CD19 immunotherapy-driven loss of CD19 expression is observed (20). Tembhare et al. demonstrated significantly lower expression of CD72 on blasts, as compared to normal hematogones (16),. In our study, in vast majority of patients CD72 showed a mid-range expression on leukemic blasts (nMFI of 2-6) and only in 11% of cases it exceeded 7 nMFI units, but its overall overexpression was present in over 70% of patients. A prevalence of positive CD72 expression on blast cells, makes it potentially useful as a key alternative marker to CD19 in patients during immunotherapy. However the utility of this marker to discriminate between blasts and normal precursor B-cells may be limited due to similarly high expression of CD72 on both cell types. Interestingly, the positive expression of CD72 was maintained at day 15 and only slightly decreased at day 33 of treatment. In order to fully prove the usefulness of CD72 in identification of blast cells after immunotherapy, it is necessary to investigate the stability of its expression on leukemic cells in patients during/after CD19-targeted therapies.

Noteworthily, a high variability of antigen expression does not exclude the utility of markers such as CD304 or CD66c for MRD monitoring. In the panel presented by the EuroFlow Consortium, CD304 was combined with CD73, and CD66c with CD123, on a single fluorescence channel, increasing the discriminative power of such marker combinations in case of aberrant expression of at least one of these antigens on leukemic blasts (28).

We also correlated the marker expression data with the occurrence of the common genetic aberrations found in pediatric BCP-ALL. Unfortunately for many of them, there were only single positive cases which hampered the credibility of statistical testing. In our study, CD66c expression on leukemic cells reached significantly higher values in patients with hyperdiploidy and in those without *ETV6::RUNX1* aberration, in line with observation previously reported by Hrusak et al. and Guillaume et al. (31, 32). However, this differential expression was no longer observed at day 15 of treatment.

Guillaume et al. also concluded that the lack of CD66c expression may be predictive of the absence of *BCR::ABL1* rearrangement. On the other hand, Yi-jun Liu et al. pointed out the high positive predictive value of CD304 antigen expression in the presence of this mutation. In our cohort, only 2 patients were *BCR::ABL1* positive, in whom we observed very high CD304 overexpression compared to the remaining patients (12 vs 3 nMFI at day 0, 16 vs 1 at MRD15) (31, 33). Furthermore, we demonstrated a significant correlation of CD86 overexpression in patients with hyperdiploidy, which was also reported by Coustan-Smith et al. and Sędek et al. (17, 18). Noteworthily, in our study this pattern was also maintained at further time points, confirming the positive correlation between CD86 overexpression and the occurrence of hyperdiploidy. We also observed significantly higher CD86 expression at diagnosis in *ETV6::RUNX1*-positive patients. Nevertheless, despite the notable differences in median at day 15, it was not statistically significant, possibly due to an excessive disparity in the numbers of cases of these two genetic groups and high standard deviations in the fusion-positive subgroup.

## 5 Conclusions

CD86 is a promising marker for MRD detection in patients with BCP-ALL. It has been proved that its expression, in majority of cases, remained positive throughout entire monitoring period. It might be considered as an additional aberrant marker for discrimination of blasts from normal B-cell precursor cells, especially in patients who demonstrate overexpression at diagnosis. We also presented a high frequency of CD72 antigen overexpression, which in the vast majority of cases remains positive throughout the treatment period considered in this study. We also demonstrated a significantly higher CD86 antigen expression in patients with hyperdiploidy, and reduced CD86 and CD66c expression in *ETV6/RUNX1*-positive patients, which persisted throughout the entire remission-inducing treatment. However, since an insufficient numbers of patients with positive aberrations including hypodiploidy, *TCF-*rearrangement, *BCR::ABL1*, *TCF3::PBX1*, *TCF3::HLF* and *IKZF1*, statistical testing could not be performed which is a limitation of this study.

## Data Availability

The raw data supporting the conclusions of this article will be made available by the authors, without undue reservation.
